# Lung adenocarcinoma patients with malignant pleural effusions in hot adaptive immunity status have a longer overall survival

**DOI:** 10.3389/fonc.2022.1031094

**Published:** 2022-10-04

**Authors:** Cheng-Guang Wu, Ruben Casanova, Fabian Mairinger, Alex Soltermann

**Affiliations:** ^1^ Institute of Pathology, University Hospital Zurich, Zurich, Switzerland; ^2^ Institute of Pathology, University Hospital Essen, Essen, Germany; ^3^ Facharzt Foederatio Medicorum Helveticorum (FMH) Pathologie, Pathologie Länggasse, Ittigen, Switzerland

**Keywords:** malignant pleural effusions, lung cancer, immune microenvironnement, immune profiling, PD-L1, prognosis

## Abstract

Malignant pleural effusion (MPE) is a common complication of lung adenocarcinoma (LADC) which is associated with a dismal prognosis. We investigated the prognostic role of PD-L1 and other immunomodulators expression in the immune compartment of MPE immune composition. MPE cytologic cell blocks of 83 LADC patients were analysed for the mRNA expression of 770 cancer-immune genes by the NanoString nCounter platform. The expression of relevant immune cell lineage markers was validated by immunohistochemistry (IHC) using quantitative pathology. The mRNA immune profiling identified four MPE patient clusters (C). C1/2 (adaptive+, hot) showed better overall survival (OS) than C3/4 (adaptive-, cold). Additionally, cold immunity profiles (adaptive-), C4 (innate+) were associated with worse OS than C3 (innate-). High PD-L1 expression was linked to the regulation of T cell activation and interferon signalling pathways. Genes of pattern recognition receptor and type I interferon signalling pathways were specifically upregulated in the long-survival (≥90 days) patient group. Moreover, immunomodulators were co-activated and highly expressed in hot adaptive immunity patient clusters, whereas *CD274* (PD-L1), *TNFRSF9* (4-1BB), *VEGFA* (VEGF-A) and *CD276* (B7-H3) were upregulated in the groups referred as cold. The patient cluster, age and PD-L1 expression were independent prognosticators for LADC MPE patients (p-value < 0.05). Our study sheds light on the variances of immune contexture regarding different PD-L1 expression and survival conditions. It revealed four distinct prognostic patient clusters with specific immune cell components and immunomodulator expression profiles, which, collectively, is supportive for future therapeutic and prognosis for cancer management.

## Introduction

Lung cancer is the leading cause of cancer-related death worldwide and lung adenocarcinoma (LADC) is its most frequent subtype with 40%. LADC often spreads to the pleural cavity, which results in a poor median overall survival of around 3 months after diagnosis (1, 2). Due to lymphatic drainage from the peripheral lung parenchyma into the pleural cavity or tumoural pleural effraction, a primary subpleural LADC may still be rather small per se. Thus, control of the pleural spread may increase overall survival significantly. However, despite the advance in chemotherapy regimens, only modest gains have been made in the long-term survival of LADC patients with malignant pleural effusion, owing to the aggressive property and complexity of the MPE microenvironment. Commonly, MPE exerts an immunosuppressive function but contains high concentrations of immune cells and cytokines due to liquid sequestration. This eventually leads to cancer progression but also offers a possibility for immunotherapy (3, 4). Moreover, heterogeneity in immune cell composition (5, 6) and cytokine expression (7–9), in MPE leads to different patients’ survival, although the mechanisms invoked therein require more elucidation. Therefore, to better understand the prognostic mechanism of immune relevant pathways and find prognosticators as well as new potential therapeutic targets for MPE LADC patients, a comprehensive investigation of the MPE immune microenvironment is desired.

We previously presented a combination of computerised immunohistochemical and transcriptional methods for MPE sample analysis to investigate the prognostic potential of immune cell composition alteration and especially immunomodulators (10, 11). We found that MPE patients with high B cells but low neutrophils to leukocytes ratio in the effusion liquids had better clinical outcomes. Moreover, MPE LADC patients were characterized by a significantly higher frequency of PD-L1 high expression compared to other MPE cancer types. By using other computational methods, several recent studies have characterised the immune infiltration features according to immune gene expression signatures in many solid tumour types with prognostic values (12–20).

Among all immunomodulators, CTLA-4 and PD-1/PD-L1 are the most actively studied ones for clinical cancer immunotherapy. Membranous expression of PD-L1 on tumour cells is a biomarker for identifying suitable NSCLC patients for PD-1/PD-L1 mAb treatment, since high expression level of PD-L1 correlated with better response rates (21, 22). Furthermore, due to its immunosuppressive function, high PD-L1 expression is also a poor prognosticator for patients without immunotherapy in many cancers, including lung and breast carcinoma as well as melanoma (23–25). Additionally, it was recently found that high PD-L1 expression resulted in worse clinical outcomes for pleural mesothelioma patients (26, 27). In contrast to the extensive pieces of literature about PD-L1 in primary solid lung carcinomas, little is known regarding its expression and prognostic role in the immune microenvironment of LADC effusion liquids (28).

Our study aimed to investigate the prognostic significance of immune system relevant pathways and immune cell composition in the malignant pleural effusions of pM1a LADC patients by performing comprehensive immune profiling of this microenvironment on formalin-fixed and paraffin-embedded cytologic cell blocks by IHC and NanoString at the protein and mRNA levels. Furthermore, the profiling was focused on the investigation of clinically relevant immunomodulators, in particular PD-L1.

## Materials and methods

### Patient cohort

Cytologic cell blocks prepared from the centrifugation sediments of 83 MPE LADC patients in the period of 2005 to 2013 were enrolled. Only cell blocks having > 20 clusters of cancer cells per whole section surface were included, and all cases were classified based on clinical data, morphology, and immunochemistry with respective markers by pathologists. The institutional review board of the University Hospital Zurich approved the study under reference number StV 29-2009-14.

### Preparation of cellblocks and cytologic microarray (TMA)

As described previously (29), the effusion liquids were centrifuged and the sediments were transferred into a microtube. Subsequently, thrombin and plasma were added for clot formation. After formalin fixation, clots were paraffin-embedded and haematoxylin-eosin (H&E) stained. From the most representative region of the donor block, two paraffin cores of 0.6 mm diameter and 3-4 mm height were taken and arrayed into a new recipient paraffin block using a tissue arrayer (Beecher Instruments).

### RNA isolation and NanoString mRNA expression analysis

Total RNA was extracted from whole sections of 75 MPE LADC cell blocks (two sections, each 5 µm thick) using the Maxwell purification system (Maxwell RSC RNA FFPE Kit, AS1440, Promega). RNA was eluted in 50 µl RNase-free water and stored at -80°C. RNA concentration was measured using a Qubit 2.0 fluorometer (Life Technologies) appertaining the RNA broad-range assay. RNA integrity was assessed using a fragment analyser (Agilent Technologies) appertaining DNF-489 standard sensitivity RNA analysis kit. 770 genes (including 40 housekeeping genes) from 24 different immune cell types were analysed with a commercially available nCounter PanCancer Immune Profiling from NanoString Technologies (Seattle, WA, USA), as per manufacturer’s instructions. Probes were hybridised to 50 ng of total RNA for 20 hours at 65°C and applied to the nCounter preparation station for automated removal of excess probe and immobilisation of probe-transcript complexes on a streptavidin-coated cartridge. Data were collected with the nCounter digital analyser by counting the individual barcodes.

Analysis and normalisation of the raw NanoString data were conducted with nSolver analysis software v 4.0 (NanoString Technologies) following the manufacturer’s recommendations. In detail, the expression values were normalised using positive controls to eliminate platform-related variation, negative controls to eliminate background effect, and 20 housekeepers to remove variation due to sample input. The data were eventually log-transformed (base 2) and ready for further analysis.

### Gene data processing

Cell type scores (13) were performed using nSolver v 4.0. Briefly, 60 marker genes for 13 immune cell populations were selected. These marker genes were specific to a single cell type and with stable expression within that cell type. Only the markers with gene expression level above the threshold of the quality control were used for cell type scores analysis. Cell scores were calculated as the average log2 normalised expression of each cell’s marker genes. The total tumour infiltrating lymphocytes (TILs) score was calculated as the average of all cell scores whose correlations with *PTRPC* (CD45) exceeded 0.6. Based on common highly expressed genes in NK cells, CD8 T cells and Tγδ, the cytotoxic cells group was defined.

Immune cell-type-specific and immunomodulator gene panels, respectively, were designed according to the literature (12, 15, 16). The immune cell-type-specific gene panel (74 genes) was used for unsupervised gene cluster analysis (ward.D). The immunomodulator gene panel was applied for gene correlation and expression analysis.

Differential gene expression analysis was performed using the R package DESeq2 (30). The p-values were adjusted for multiple comparisons by using the false discovery rate (FDR, Benjamini-Hochberg method). Genes that had both a 2-fold change in expression between the compared groups, and an FDR less than 0.05 were used in downstream analyses.

Differentially expressed genes were mapped to the Gene Ontology (GO) term. Analysis and visualisation of GO terms associated with differentially expressed genes were performed using ClueGO (http://apps.cytoscape.org/apps/cluego) (31) a plug-in for Cytoscape (3.7.1). The immune system related pathways are functionally grouped and interconnected based on the kappa score. The size of the nodes shows the term significance after the Benjamini-Hochberg correction. Only terms with corrected p-value ≤ 0.05 were considered. Homo Sapiens Immune System Process (GO release 24.06.2018) was used as a background annotation database to identify pathways that are overrepresented in a phenotype.

### Immunohistochemistry and scoring

For IHC analysis 3 μm thick sections were cut. Laboratory developed assays were tested on a multi-tissue microarray for clone E1L3N (Cell Signaling Technology, dilution 1:100). Anti-PD-L1 antibody clone SP263 (Ventana, prediluted) was used according to the manufacturer’s recommendation. IHC using antibodies against CD3 (mature T-cells), CD4 (helper T-cells), CD8 (cytotoxic T cells, Tc cells), CD20 (B cells), CD45 (leukocytes), CD68 (macrophages), myeloperoxidase (MPO, neutrophilic granulocytes) were performed as previously described (10). IHC stainings were performed on a Benchmark Ultra platform (Ventana) with protocols used for routine diagnostics. Afterward, the stained slides were scanned by a high-resolution scanner (Nanozoomer Digital Pathology). All primary antibodies used for IHC analysis were listed (**Supplementary Table S1**).

PD-L1 immunoreactivity was semi-quantitatively scored by experienced pathologists. The scoring was dichotomised into low (0 to 49%) and high (≥50%), taking into account only unequivocal membranous staining of tumour cells. PD-L1 (clone E1L3N) high and low expressing groups were compared using cancer immune gene expression data (differential expression analysis, the 730 gene panel).

For the immune cell quantification, the ratios of CD3, CD4, CD8, CD20, CD68 and MPO-positive immune cells were calculated using QuPath (32), an open-source software for quantitative pathology (Queen’s University, Belfast), as follows: (positive cell count/mm^2^)/(CD45+ cell count/mm^2^). Briefly, after adjusting the RGB values of the TMA image, the positive cell detection algorithm using optical density sum was applied for the representative core. Parameters such as sigma, threshold, and background radius were further adjusted until decent cell detection was achieved. Eventually, this fixed detection algorithm was automatedly applied to quantify the tissue area, total cell number, and positive cell number for the rest of the TMA cores.

### Statistical analysis

All statistical analyses were performed on SPSS software (version 23, IBM) or environment R (version 3.4.2, R Core Team). Overall survival (OS) was defined as the period from the date of first MPE diagnosis to patients’ death and was computed using the Kaplan-Meier method and log-rank tests. Cox proportional regression analysis was performed to analyse the prognostic influences of gene clusters, PD-L1 and other clinico-pathologic parameters. The Shapiro-Wilks test was applied to test for the normal distribution of each data set. Based on the results, for dichotomous variables either the Wilcoxon Mann-Whitney rank sum test (non-parametric) or the two-sided Student’s t-test (parametric) was used. For ordinal variables with more than two groups, either the Kruskal-Wallis test (non-parametric) or ANOVA (parametric) was used to detect group differences. Correlation matrices were created with Pearson’s correlation. P-value was adjusted for multiple comparisons by using the false discovery rate (FDR, Benjamini-Hochberg method). A p-value ≤ 0.05 was considered significant.

## Results

### Cohort description

In our cohort of 83 MPE LADC patients (median age of 70 years), the estimated median overall survival (medOS) from the diagnosis of malignant effusion was 107 days (**Table 1**). 20% of the tumours were highly expressing PD-L1. Three inconsistently scored cases were found between antibody clone E1L3N and SP263 (two cases E1L3N high, one case SP263 high). Representative IHC staining images are presented (**Supplementary Figure S1**).

**Table 1:** Cohort description and PD-L1 immunohistochemistry.

	n total = 83	Median (range)
OS (days)		107 (44-170)
Age (years)		70 (29-93)
		N (%)
Sex	male	45 (54%)
	female	38 (46%)
Other metastases	with	28 (54%)
	without	24 (46%)
Chemo before diagnosis	yes	22 (39%)
	no	34 (61%)
PD-L1 E1L3N	high	17 (21%)
	low	65 (79%)
PD-L1 SP263	high	16 (20%)
	low	65 (80%)

OS, overall survival; CI, confidence interval.

### Immune gene clusters correlate with patients overall survival

**Figure 1:**
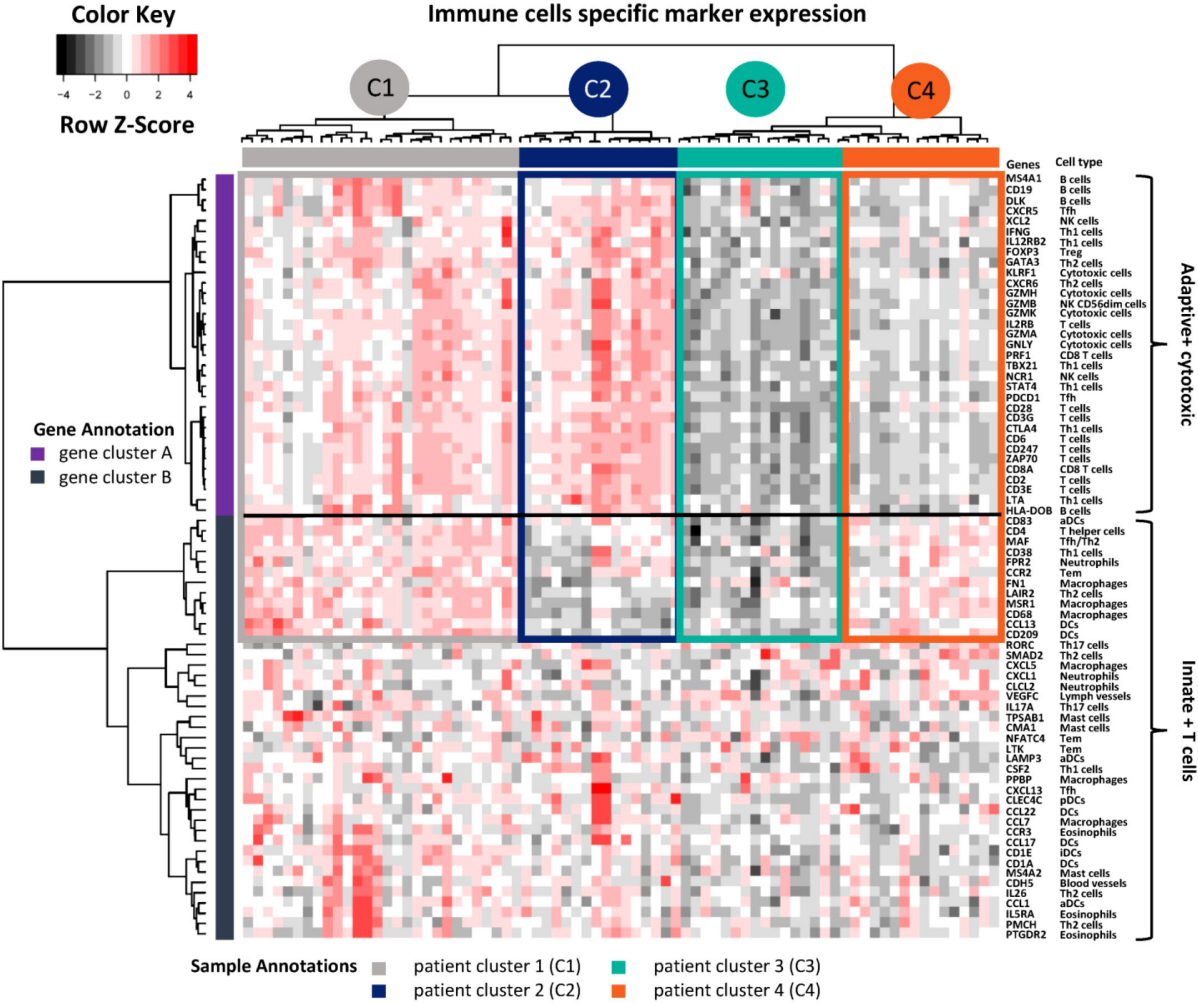
Immune cell type-specific gene expression. Unsupervised hierarchical clustering was performed for 74 genes from 75 LADC patient MPE samples. High expressed genes are indicated in red, low ones in black. The corresponding immune cell types are shown on the right side. Genes were further annotated as clusters adaptive + cytotoxic versus innate + T cells. Patients were unsupervised grouped into four clusters (C1 to C4) and annotated with different colours. Th1, T helper cells 1; NK, natural killer cells; Tem, T effector memory; Tfh, T follicular helper cells; Tγδ, T gamma delta; Treg, regulatory T cells; DCs, dendritic cells; iDCs, immature DCs; aDCs, activated DCs; pDCs, plasmacytoid dendritic cells.

After quality control, mRNA screening was performed for 75 patient MPE samples using a panel of 730 cancer-immunity relevant genes. For immune cell-type-specific gene analysis, genes were clustered into two main groups (**Figure 1**): gene cluster A contains markers of adaptive and cytotoxic immune cells, including B cells, T cells including interferon-gamma and CTLA4-expressing T helper cells 1 (Th1) as well as CD8 T cells, and natural killer (NK) cells. Gene cluster B denotes innate immune cells such as macrophages, dendritic cells (DCs) and neutrophils as well as other subtypes of T cells such as CD38 Th1, Th2, T effector memory (Tem), and T follicular helper (Tfh) cells). Patients were then separated into hot and cold groups according to the expression level of gene cluster A. In the hot expression group, patients were further grouped into patient cluster 1 (C1, adaptive+, innate+) and 2 (C2, adaptive+, innate-) according to the expression levels of gene cluster B. Similarly, patient cluster 3 (C3, adaptive-, innate-) and 4 (C4, adaptive-, innate+) were sub-clustered from the cold group.

**Figure 2:**
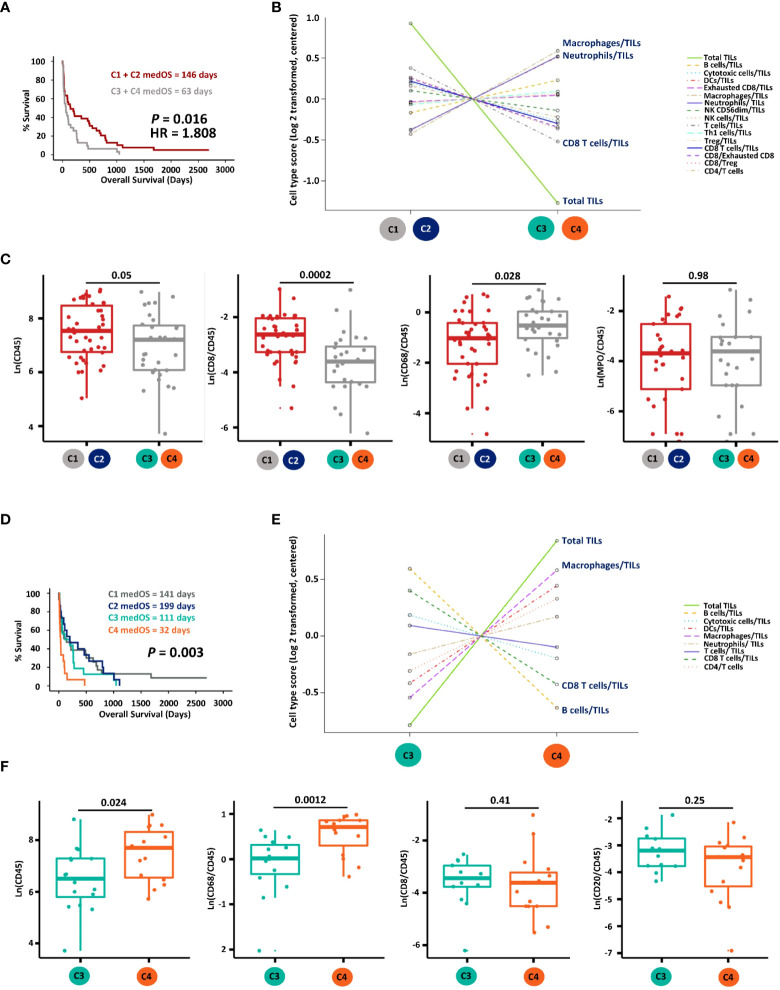
Prognostic and immune cell composition analyses of the four gene clusters. **(A)** Kaplan-Meier survival analysis of hot (cluster C1 + C2) versus cold (cluster C3 + C4) MPE LADC patients. **(B)** Immune cell type score comparison between hot and cold MPE LADC tumours on mRNA level. **(C)** Immune cell component comparison between hot and cold MPE LADC tumours according to IHC quantifications. Immune cell markers with the highest two (macrophages/TILs and neutrophils/TILs) and lowest two (CD8 T cells/TILs and total TILs) immune cell type scores were selected for IHC validation. **(D)** Kaplan-Meier survival analysis of all patient clusters (C1 to C4). **(E)** Immune cell type comparison of gene clusters C3 versus C4 on mRNA level. **(F)** Immune cell type comparison of gene clusters C3 versus C4 according to IHC quantifications. Immune cells markers with the highest two (macrophages/TILs and total TILs) and lowest two (CD8 T cells/TILs and B cells/TILs) scores were selected for IHC validation. For Kaplan-Meier survival analysis, the p-value (P) of log-rank tests is indicated. medOS, median overall survival; TILs, tumour infiltrating lymphocytes.

We then addressed the prognostic relevance of these four immune clusters. Patients with hot MPE (C1 + C2) presented significantly longer OS compared with the cold group (C3 + C4) (**Figure 2A**). The mRNA-based cell type score analysis revealed that hot MPE contained higher TILs and a higher CD8+ T cell/TILs ratio, while cold samples had more macrophages and neutrophils (**Figure 2B**). These results were corroborated by corresponding IHC scores using CD45 for TILs, CD68 for macrophages and MPO for neutrophils (**Figure 2C**), and the detailed digitalized image quatification methodology was descriped in our previous publication (10). Furthermore, when comparing the survival curves, we found that C4 patients had the worst OS with a median of only 32 days (**Figure 2D**). Compared to C3, C4 MPE had higher TILs and higher macrophages/TILs but lower CD8/TILs and B cells/TILs ratios according to mRNA-based cell type scoring as well as IHC quantification (CD20 for B cells) (**Figures 2E, F**).

### High tumour cell PD-L1 expression is prognostic and immunogenic

**Figure 3:**
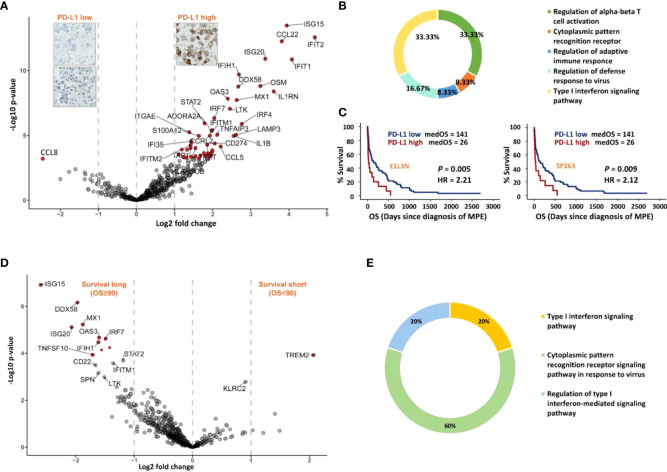
Immune gene expression analysis according to MPE LADC PD-L1 expression (tumour cells) and patient survival. **(A)** Differential 730 genes expression analysis. Genes with twice fold expression change and FDR less than 0.05 were marked in red. **(B)** Chart showing the most important cell biological pathways upregulated in the PD-L1 high group from Gene Ontology (GO) analysis. **(C)** Kaplan-Meier survival analysis for patients with PD-L1 high versus low expressing tumours, using clones E1L3N and SP263. The P-value (P) of the log-rank test is indicated. **(D)** Differential gene expression (730 genes) analysis of patients with long and short-survival (cut-off = median OS, 90 days), respectively. Genes with 2 times fold expression change and FDR < 0.05 were marked in red. **(E)** Chart showing the most significant terms in the long-survival group (GO analysis). Only terms with corrected p-value < 0.05 were shown.

As shown in the differential expression analysis (**Figure 3A**), the PD-L1 high group showed increased expression of specific genes with an FDR < 0.05 and a log2 fold change > 1. In particular, immune checkpoints *CD274* (PD-L1), *TIGIT* (T cell immunoreceptor with Ig and ITIM domains, TIGIT) and *ADORA2A* (adenosine A2a receptor) and immune co-stimulatory genes *ICOS* (CD278, inducible T-cell co-stimulator, ICOS) and *CD27* (CD27, TNFRSF7) were associated with high expression of the PD-L1 protein. All genes differentially expressed in MPE containing PD-L1 high expressing LADC tumour cell clusters were subsequently mapped to Gene Ontology (GO), and relevant immune system pathways, called terms, were functionally grouped and interconnected (**Figure 3B**, **Supplementary Figure S2**). Briefly, increased gene expression in high PD-L1 MPE LADC involved particularly the regulation of alpha-beta T cell activation (one-third of significant terms) and type I interferon signalling (another third of significant terms). In addition, immune cell type analysis by IHC showed that MPE with high PD-L1 LADC had higher exhausted CD8 T cell expression but lower expression of innate immune cells such as DCs and macrophages (**Supplementary Figure S3**). For survival analysis, high expression (≥ 50% positive tumours cells) of PD-L1 correlated with significantly shorter patient’s survival (p-value < 0.01, **Figure 3C**).

### Pathways of cytoplasmic pattern recognition receptor and type I interferon signalling are upregulated in long-surviving patients

To compare the immune profiles between MPE LADC patients with long and short-survival, respectively, we performed differential gene expression and functional annotation enrichment analyses using a median time of 90 days as the survival cut-off. Patients with longer survival presented a more immunogenic context, while short-survival patients had only *TREM2* (triggering receptors expressed on myeloid cells 2, TREM2) preferentially expressed (**Figure 3D**). According to GO analysis, all these long patient survival associated genes were involved in pathways of cytoplasmic pattern recognition receptor signalling and/or type I interferon signalling (**Figure 3E**, **Supplementary Figure S4**). In particular, overexpressed genes *IFIH1* (melanoma differentiation-associated protein 5, MDA-5) and *DDX58* (Retinoic acid-inducible gene I, RIG-I) are the cores of RIG-I-like receptors (retinoic acid-inducible gene-I-like receptors, RLRs), which belongs to RNA-sensing pattern recognition receptors (PRRs). In addition, the *IRF7* molecule (interferon regulatory factor 7, IRF7) is downstream of type I interferon signalling playing a pivotal role in initiating multiple anti-cancer immune responses.

### Correlation of immunomodulators with patient clusters

**Figure 4:**
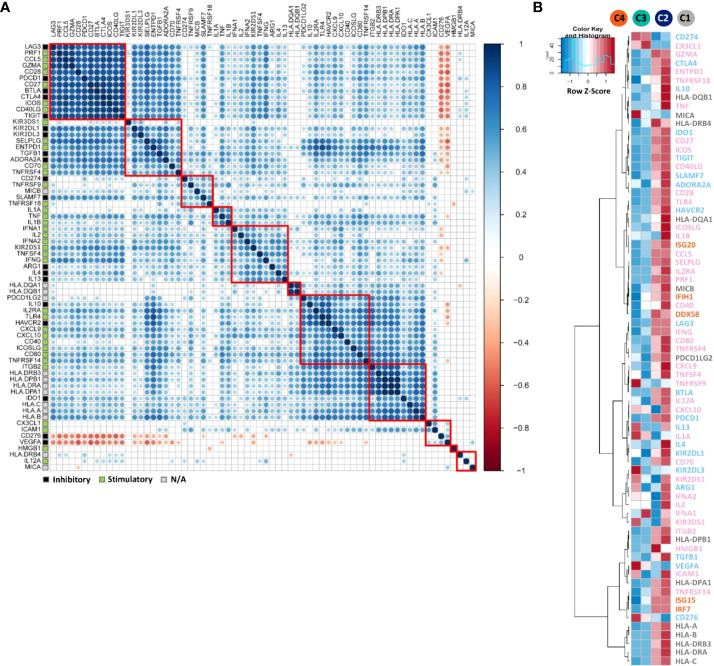
Correlation and expression analyses of immunomodulator genes. **(A)** Pearson correlation matrix showing the clustering and correlations of 68 immunomodulator genes. The correlation matrix was subjected to unsupervised hierarchical clustering using Euclidean distance measurement. Blue dots illustrate positive correlations, white ones no correlation (FDR ≥ 0.01), and red negative correlations. Bigger dot size indicates a smaller p-value of the correlation. Red squares indicate the individual clusters. Different gene modulatory functions were annotated in coloured squares on the left. Black indicates the inhibitory function, green stimulatory and grey modulatory function not known (N/A). **(B)** heatmap indicating the average mRNA expression of 68 immunomodulator genes together with five genes from long-survival relevant pathways such as RIG-1-like receptors (RLRs) and type 1 interferon pathways, among the four patient clusters C1-C4. mRNA expression data were normalised and unsupervised clustered using Euclidean distance measurement. For the gene expression level, relatively high level expressions are in red, low level in blue. On the right side of the figure, inhibitory gene names were marked in light blue, stimulatory ones were in pink, unknown modulatory function genes in grey and RLRs and type I interferon pathway relevant genes in orange.

We then extracted 68 immunomodulator genes, including most immune checkpoints and immune co-stimulator molecules from the 730-gene panel. These modulators play key roles in either anti- or pro-tumoural immune system actions, and most of them are targetable by agonists or antagonists being evaluated in clinical oncology (33). Even though immune checkpoints and immune co-stimulators may exert opposite functions, their gene expression levels were found to be positively correlated as shown in **Figure 4A**. For instance, immune checkpoints *LAG-3* (Lymphocyte-activation gene 3, LAG-3), *PDCD1* (PD-1), *BTLA* (B- and T-lymphocyte attenuator, BTLA), *CTLA-4 and TIGIT* were strongly correlated with co-stimulatory molecules *CD28*, *CD27*, *ICOS* and *CD40LG* (CD40 ligand). Moreover, *CD274* (PD-L1) clustered closely with the co-stimulatory marker *TNFRSF9* (4-1BB). In contrast, in this set of 68 genes, only *CD276* (B7-H3) and *VEGFA* were negatively correlated with the other genes.

The mean gene expression levels of immunomodulators and long patient’s survival relevant pathway genes (RLRs and type I interferon pathways) were compared (**Figure 4B**). Most of the immunomodulator genes and all long-survival relevant pathway genes (**Figure 3D**) were highly expressed in hot clusters C1 and C2. However, even defined as the cold immune microenvironment, genes such as *CD274* (PD-L1), *TNFRSF9* (4-1BB), *VEGFA, CD276* (B7-H3) and *KIR2DL3* (Killer cell immunoglobulin-like receptor 2DL3, KIR2DL3) were particularly highly expressed in C3 and/or C4.

### Prognostic MPE LADC patients

Finally, the above-studied markers were submitted to univariable and multivariable Cox regression analyses together with relevant clinico-pathologic parameters. Age above 70 years, gene clusters and high expression of PD-L1 were associated with decreased overall survival (**Table 2**) and remained independent in the following multivariate analysis.

**Table 2:** Univariable and multivariable Cox regression analysis of prognostic parameters.

	p-value	Univariale HR (95% CI)	p-value	Multivariale HR (95% CI)
Age (> 70 years *vs*. ≤ 70 years)	0.012	1.810 (1.142 - 2.870)	0.001	2.513 (1.457 - 4.334)
Sex (male *vs*. female)	0.292	0.784 (0.499 - 1.232)		
Other metastasis (with *vs*. without)	0.415	1.266 (0.718 - 2.233)		
Chemo before diagnosis (yes *vs*. no)	0.293	1.356 (0.769 - 2.392)		
Patient cluster (C4 *vs*. C3 *vs*. C1+C2)	0.001	1.699 (1.234 - 2.339)	0.003	1.648 (1.180 - 2.301)
PD-L1 (high *vs*. low)	0.006	2.214 (1.254 - 3.912)	0.001	3.316 (1.661 - 6.617)

HR, hazard ratio.

## Discussion

The immune microenvironment contributes to tumour development, migration, and progression, eventually resulting in different survival outcomes for individual cancer patients. In this study, we performed comprehensive immune profiling of LADC MPE samples using digitalised transcriptional and immunohistochemical approaches on formalin-fixed paraffin-embedded (FFPE) cytologic cell blocks. To the best of our knowledge, this is the first MPE LADC study with a detailed description of its immune landscape.

Using a comprehensive immune system gene expression panel, we identified four distinctive gene clusters corresponding to specific immune cell compositions in the MPE LADC liquids with associated respective immunomodulator expressions. These profiles correlated with different patient’s prognosis, suggesting the possibility of individualised management and therapeutic strategy. Furthermore, high expression of the therapeutic and prognostic molecule PD-L1 was proved to have a strong correlation of the increased expression of genes belongs to the T cell activation regulation pathways, together with the co-stimulation of many other immunomodulators (mRNA level). Of note, genes in pattern recognition receptor (*IFIH1*, *DDX58*, and *IRF7*) and type I interferon signalling pathways (*ISG15*, *ISG20*, *IRF7*, etc.) were highly expressed in long-survival patients, which might be further exploited for potential novel MPE therapeutic targets.

Immunotherapy is a novel and potent anti-tumour treatment method; thus, the characterisation of tumour immune microenvironment biology is of high clinical relevance for patient’s prognostic stratification and individualised therapies (34, 35). Because of the sequestered character of effusion liquids confined in body cavities, a high concentration of immune cells and immunosuppressive cytokines can be expected. Comprehensive characterisation of MPE’s liquid immune microenvironment might help stratify patients who could benefit from immunotherapy (3). In this study, we adapted an immune cell type-specific gene panel based on recently published data. Genes were selected when exclusively expressed in a single immune cell type under the condition of presenting a stable expression within that cell type (12, 13, 15–17, 19).

This allowed identifying gene sets by unsupervised clustering annotated as adaptive plus cytotoxic cells versus innate plus T cells. Together with the cell type score and IHC quantification results, we expanded the concept of hot and cold tumours by not only considering the T cells infiltration but also including other adaptive and cytotoxic immune cells. The corresponding hot tumour patients were found to have a better prognosis compared to the cold ones, which might be relevant for MPE LADC patient management. Additionally, cold tumour patients were further stratified according to the innate immune cells infiltration, namely C3 (innate-) and C4 (innate+). Surprisingly, patients in the innate+, adaptive- cluster (C4) presented the worst survival among all patient clusters but not the extremely cold cluster C3 (adaptive-, innate-). This result might be explained by immunosuppressive and inflammatory pro-tumour effects of tumour-associated macrophages and neutrophils (36, 37), however single gene or gene signature analysis was not suitable to accurately identify macrophages (M1/M2), neutrophils (N1/N2) and DC subtypes. Our previous study also demonstrated the negative prognostic influence of neutrophils in MPE patients (10).

From the therapeutic perspective, combinational immunotherapy is a reasonable option due to the fact that most of these targetable immunomodulatory molecules were co-activated (**Figure 4A**), which was also reported by other studies (38–40). A similar co-activation trend of *PD-L1* with other immunomodulators (*TIGIT, ADORA2A and ICOS*) was seen from the differential expression analysis grouped according to the PD-L1 IHC scoring result (**Figure 3A**). Likewise, current clinical research pays more attention to bi-specific and tri-specific antibodies development as well as combination therapy, e.g. using anti-PD-L1 together with anti-TIGIT antibodies for lung cancer patients (NCT04294810) in order to obtain, on the one hand, better efficacy, on the other hand, to solve treatment resistance problems (41, 42). Inconsistent with most other positively correlated genes, *CD276* (B7-H3) and *VEGFA* were negatively associated with other genes. As a poor prognosticator for many malignancies, B7-H3 was overexpressed in tumour cells and might exert a suppressive function for both adaptive and innate immune responses according to our findings and other studies (43). Moreover, B7-H3 was overexpressed in the blood vessels of human tumours (44) and promoted angiogenesis through the enhancement of VEGF secretion (45). Intriguingly, together with *CD274* (PD-L1), *CD276* (B7-H3) and *VEGFA* were both highly expressed in the cold tumours, which could be further investigated as novel immunotherapy targets for such patients.

In addition to the immunomodulators, our finding emphasised the prognostic significance of a pathway belonging to the primitive immune system, named pattern recognition receptors (PRRs). The activation of PRRs by virus RNA or cancer cells (46, 47) results in upregulation of the type I interferon pathway (mainly from the host DCs) which consequently initiates an antigen-specific adaptive immune response *via* activation of DCs (especially conventional DCs (48)) and CD8 + T cells. It also enhances NK cells cytotoxicity and mediates tumour elimination (49). Additionally, PRRs together with the type I interferon pathway were reported as intrinsic tumour suppressors, which facilitated cancer cell lysis and apoptosis (46, 47, 50). More importantly, in our cohort, genes belonging to RIG-I like receptor in PRRs and type I interferon signalling pathways (*IFIH1*, *DDX58*, *ISG15*, *ISG20* and *IRF7*) were exclusively overexpressed in the long-survival patient group and highly expressed in hot tumours, offering a promising approach for MPE cancer therapy by triggering these natural antiviral responses. Of note, high expression of type I interferon pathway genes represented better immune stimulation and activation, even though high PD-L1 expression also activated interferon pathways. Among patients showing high PD-L1 expression, the activation of the type I interferon pathway may not be the dominant factor for patient clinical outcomes, and further study needs to be done to elucidate this hypothesis. In the short-survival patient group, only the gene *TREM2* was differentially expressed. As a negative immune regulator, the expression level of the triggering receptor expressed on myeloid cells-2 (TREM2) was found inversely correlated with patient prognosis in gastric cancer (51, 52).

There are some limitations of our study. Our retrospective cohort did not include the clinical information such as patient performance status and mutation status due to the historical data incompletion for the survival analysis which may add values to our conclusion. Further functional verification for our proposed therapeutic targets and M1/M2 macrophages, N1/N2 neutrophils and DCs subpopulation validation in terms of their prognostic potential are essential for the better understanding and interpretation of MPE onco-immunity, which will be investigated in further studies.

## Conclusions

In summary, our investigation of the MPE LADC immune landscape demonstrates the clinically relevant immunogenic potential of PD-L1, and the significance of cytoplasmic pattern recognition receptor and type I interferon signalling pathways in the tumour suppressive process. Moreover, our study identified four gene clusters with different prognostic values for patient’s overall survival, specific immune components and diverse immunomodulator expression profiles, which provides practical information for predicting disease outcomes and investigating new therapeutic strategies for MPE LADC patients.

## Data availability statement

The original contributions presented in the study are included in the article/**Supplementary Material**. Further inquiries can be directed to the corresponding authors.

## Ethics statement

This study was reviewed and approved by The institutional review board of the University Hospital Zurich under reference number StV 29-200914. The patients/participants provided their written informed consent to participate in this study.

## Author contributions

CW: Conceptualization, Methodology, Investigation, Formal analysis, Writing - original draft, Writing - review and editing. RC: Data curation, Investigation, Writing - review and editing. FM: Formal analysis, Methodology, Investigation. AS: Resources, Supervision, Conceptualization, Funding acquisition, Writing - review and editing. All authors contributed to the article and approved the submitted version.

## Funding

CW is supported by a grant from the China Scholarship Council (reference number 201506240116). AS has obtained grants from the Swiss Cancer League (reference number F-87701-31-01) and the Swiss National Science Foundation Systems (reference number M-87704-01-02).

## Acknowledgments

The authors thank Susanne Dettwiler and Fabiola Prutek (University Hospital Zurich) for their technical assistance.

## Conflict of interest

The authors declare that the research was conducted in the absence of any commercial or financial relationships that could be construed as a potential conflict of interest.

The handling editor LB declared a past co-authorship/collaboration with the author FM.

## Publisher’s note

All claims expressed in this article are solely those of the authors and do not necessarily represent those of their affiliated organizations, or those of the publisher, the editors and the reviewers. Any product that may be evaluated in this article, or claim that may be made by its manufacturer, is not guaranteed or endorsed by the publisher.
