# Supplementary material for: Lung adenocarcinoma patients with malignant pleural effusions in hot adaptive immunity status have a longer overall survival
Source: Front Oncol. 2022 Oct 4;12:1031094. doi: 10.3389/fonc.2022.1031094 (PMC9577289; doi:10.3389/fonc.2022.1031094)
Supplement: Supplementary file 1 [file DataSheet_1.docx]

**Nanostring gene test datasets**

**The names of the repository:** **zenodo**

**Accession number: DOI: 10.5281/zenodo.7032262**

**Name: Malignant pleural effusion LADC immunity 700 gene dataset**

**Supplementary Figures**


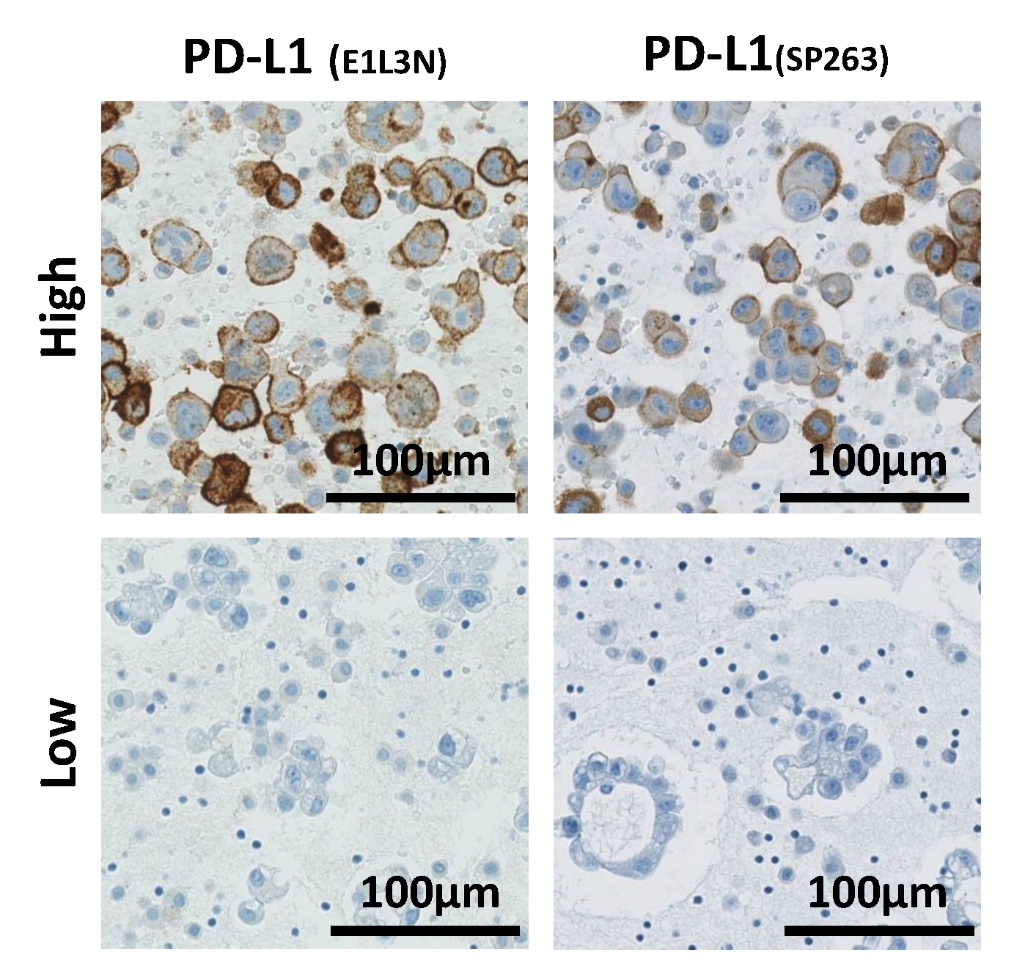


**Supplementary Fgure S1 Examples for PD-L1 (clones E1L3N and SP263) high and low expression in MPE samples.** Scale bar = 100µm.


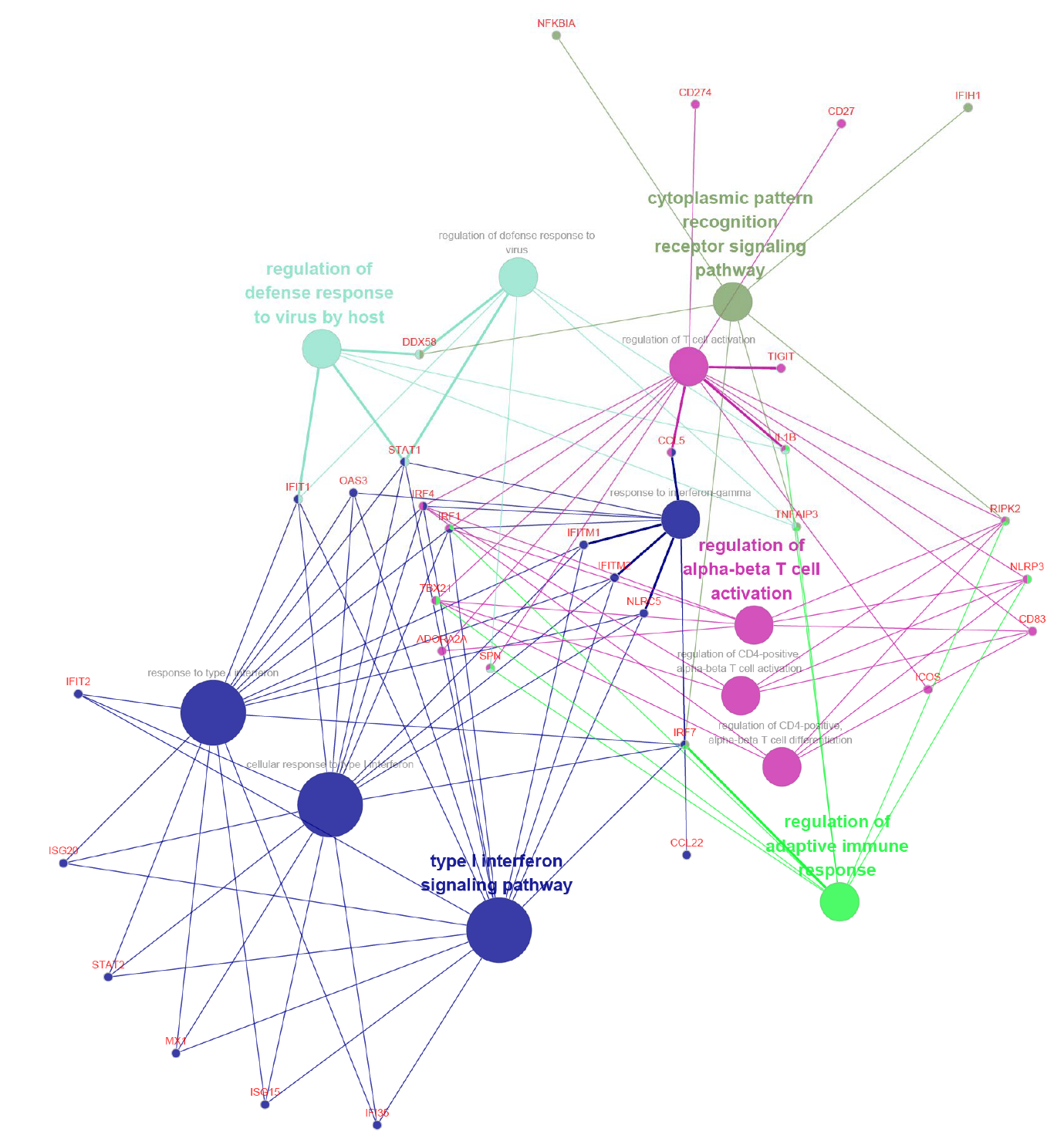


**Supplementary Figure S2.** **ClueGO analysis of significantly differentially expressed genes in the PD-L1 high expression group.**

According to figure 3A result (significantly differentially expressed genes), the immune system relevant gene ontology terms are shown as functionally grouped nodes in an interconnected network-based on kappa score level. The sizes of the nodes reflect the enrichment significance of the terms (Benjamini-Hochberg correction).


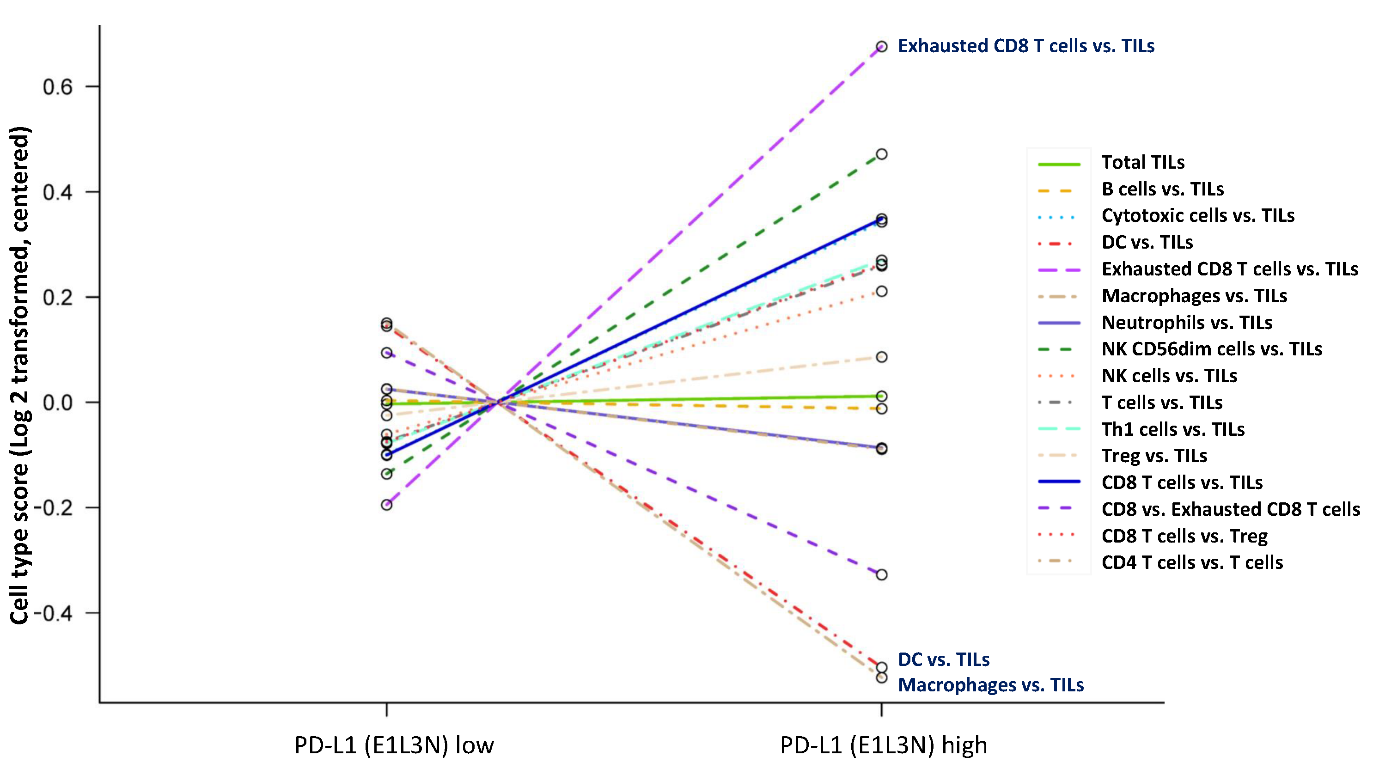


**Supplementary Figure S3. Comparison of immune cell type scores by mRNA level for low versus high PD-L1 (protein) expressing LADC in MPE.** Data were log2 transformed and cantered. Abbreviations: T helper cells 1 (Th1), natural killer cells (NK), regulatory T cells (Treg), dendritic cells (DC), tumour infiltrating lymphocytes (TILs).


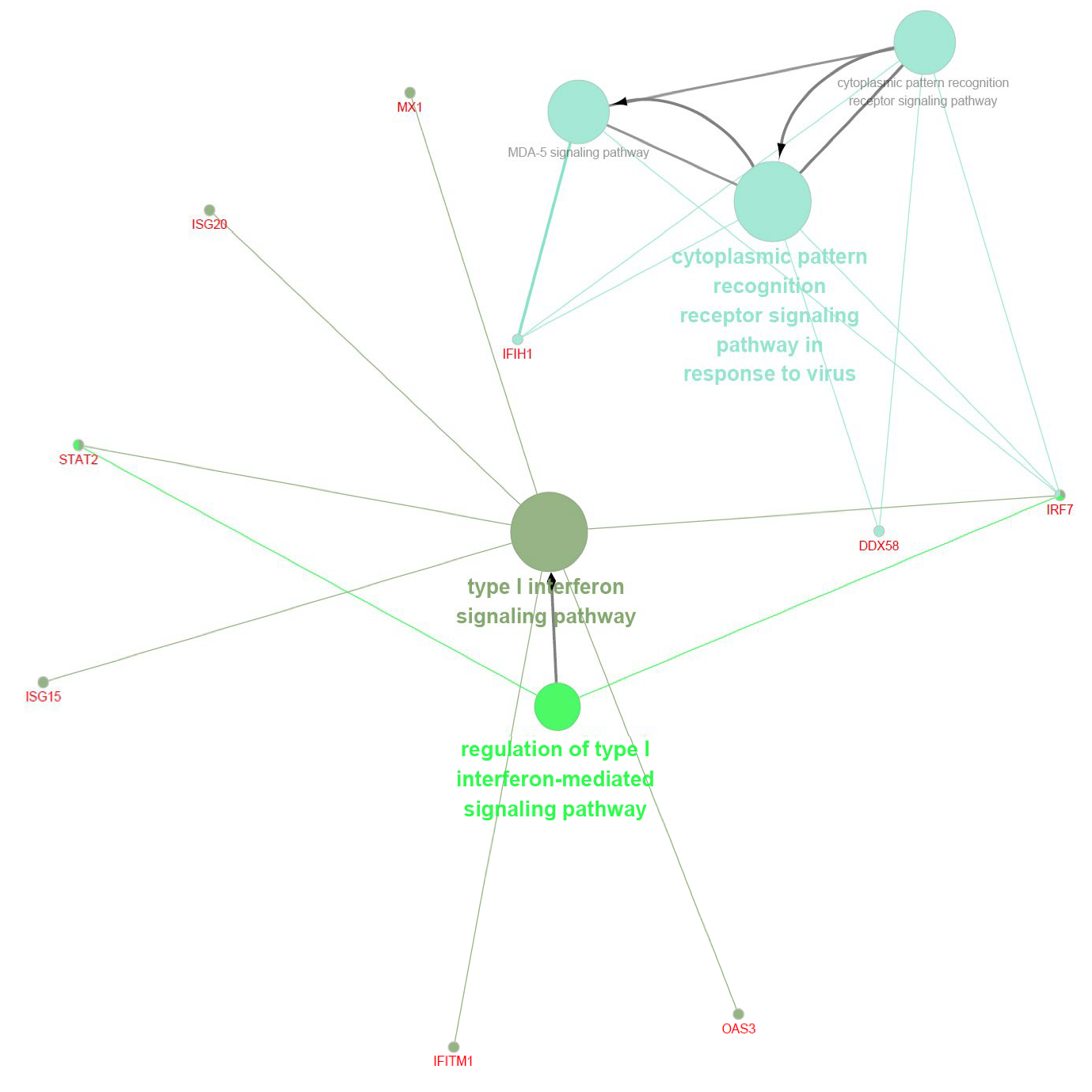


**Supplementary Figure S4. ClueGO analysis of upregulated genes in the long-survival patient group.**

According to the result of figure 3D (significantly differentially expressed genes), the immune system relevant gene ontology terms were shown as functionally grouped nodes in an interconnected network-based on kappa score level. The sizes of the nodes reflect the enrichment significance of the terms (Benjamini-Hochberg correction).

**Supplementary Table**

**Supplementary Table S1. List of antibodies used for immunohistochemistry.**

| **Antibody** | **Type** | **Clone** | **Dilution** | **Producer** |
| --- | --- | --- | --- | --- |
| CD3 | monoclonal | SP7 | 1:200 | Thermo Fisher |
| CD4 | monoclonal | SP35 | prediluted | Ventana-Roche |
| CD8 | monoclonal | C8/144B | 1:100 | DAKO A/S |
| CD20 | monoclonal | L26 | prediluted | Ventana-Roche |
| CD45 | monoclonal | PD7/26 + 2B11 | 1:250 | DAKO A/S |
| CD68 | monoclonal | PG-M1 | 1:50 | DAKO A/S |
| MPO | polyclonal |  | 1:200 | NeoMarkers/Lab Vision Corporation |
| DC-LAMP | monoclonal | 1010E1.01 | 1:100 | Novus Biologicals |
| PD-L1 | monoclonal | SP263 | prediluted | Ventana-Roche |
| PD-L1 | monoclonal | E1L3N | 1:100 | Cell Signaling Technology |
